# Assessment of the influence of the patient’s inflammatory state on the accuracy of a haptoglobin selected reaction monitoring assay

**DOI:** 10.1186/1559-0275-11-38

**Published:** 2014-11-01

**Authors:** Olivier Lassout, Denis Hochstrasser, Pierre Lescuyer

**Affiliations:** Department of Genetic and Laboratory Medicine, Geneva University Hospitals, Geneva, Switzerland; Department of Human Protein Sciences, Faculty of Medicine, Geneva University, Geneva, Switzerland

**Keywords:** Inflammation, Haptoglobin, Protease inhibitor, Quantitative proteomics, Selected reaction monitoring, Trypsin digestion

## Abstract

**Background:**

The use of targeted LC-MS/MS methods for protein quantitation in clinical laboratories implies a careful evaluation of potential sources of analytical interference. In this study, we investigated whether inflammation, which is associated with both the release of proteolytic enzymes and increased expression of acute phase protease inhibitors, is affecting the accuracy of a haptoglobin selected reaction monitoring (SRM) assay.

**Results:**

A SRM assay was developed and used to quantify haptoglobin in 57 human serum samples. The SRM assay had CVs (n = 6) of 12.9% at 698 mg/L and 11.8% at 1690 mg/L. Results of the SRM assay were compared to those of a commercial immunonephelometric test. Passing-Bablok regression gave a proportional bias of 0.92 (95% CI: 0.82 to 1.04) and a constant bias of 75.40 (95% CI: −71.09 to 251.04), indicating that SRM and immunonephelometric assays provided comparable results. We then investigated whether the accuracy of the SRM assay was influenced by the patient’s inflammatory state by assessing the relationship between the serum CRP concentration and the bias between the two methods. No correlation was found between the SRM/immunoassay bias and the CRP concentration (Pearson correlation coefficient r = 0.0898).

**Conclusions:**

These data indicate that neither the release of proteolytic enzymes nor the increased level of protease inhibitors occurring during inflammation processes have a significant impact on the haptoglobin SRM assay accuracy. Such studies provide important information about potential sources of analytical interferences in protein SRM assays.

**Electronic supplementary material:**

The online version of this article (doi:10.1186/1559-0275-11-38) contains supplementary material, which is available to authorized users.

## Background

Thanks to the development of instruments and protocols, the use in clinical laboratories of liquid chromatography coupled to tandem mass spectrometry (LC-MS/MS) is expanding. A combination of factors explains this trend. First, very high analytical performances (sensitivity, scan speed) can be achieved with relatively simple and entry-level instruments, such as ion trap or triple-quadrupole mass spectrometers. Second, improvement of the robustness of LC-MS/MS systems and the development of user-friendly software interfaces make the use of LC-MS/MS for routine applications by a non-specialized team easier. Third, the availability of commercial reagents, including HPLC columns, mobile phases, calibrators, and internal standards, together with dedicated support from vendors for clinical applications greatly simplifies the implementation of a new MS assay. All these factors have contributed to the democratization of the use of LC-MS/MS methods in clinical laboratories. Reports from external quality schemes shows that LC-MS/MS is becoming a major player in some important clinical areas, such as therapeutic drug monitoring of immunosuppressants or measurement of 25-OH-vitamin D and steroid hormones.

It is noteworthy that MS protein assays do not yet follow this trend despite an indubitable and sustained interest from the clinical chemistry community [1, 2]. MS assays may indeed provide a number of advantages over immunoassays, which are classically used for protein quantification in clinical laboratories, particularly in terms of multiplexing capabilities and analytical specificity. Hoofnagle and Werner have thus described different situations where immunoassay limitations could be overcame by the use of MS. This includes the lack of inter-assays standardization due to the use of different antibodies recognizing different epitopes and the analytical interferences related to the presence of autoantibodies or anti-reagent antibodies in patients’ serum [3]. Moreover, numerous selected reaction monitoring (SRM) protein assays have been developed for the quantification of proteins of clinical interest in serum or urine. Some of these studies included a complete analytical validation and a comparison with a reference method to fulfill criteria used in laboratory medicine for method qualification. The list includes albumin [4, 5], alpha-1-antitrypsin [6], apolipoproteins A1 and B [7], parathyroid hormone [8, 9], thyroglobulin [10, 11], and troponin I [12]. However, at the present time, the application of these methods in a clinical setting, if any, is restricted to one or a few laboratories. One reason is probably the complexity of the workflow used for sample preparation, involving trypsin digestion and, often, an immunoaffinity step for target peptide enrichment [8–12]. The ability to run such assays on a routine basis remains then restricted to a few specialized laboratories with dedicated instruments and team. In addition, these SRM protein assays have their own specific problems in term of analytical interferences. Trypsin digestion, in particular, was shown to be a major factor of analytical imprecision [13]. Peptidase activities present in biological samples may also be a source of analytical errors by degrading peptides generated from the target protein or heavy isotope-labeled peptides used as internal reference standards [14, 15]. Therefore, the biological and technical factors that can impact the performances of SRM protein assays must be carefully assessed before considering applying such methods in routine clinical practice.

According to what has been shown regarding the critical influence of trypsin digestion and peptide stability on SRM protein assay performances, inflammation, which is present in a wide range of disease states, represent a particular concern in terms of analytical interferences. Inflammation is indeed associated first with the release of various proteolytic enzymes [16] that may affect protein and peptide stability in serum samples. Second, inflammation is associated with an increased expression of acute phase proteins with antitrypsin activities, such as alpha-1-antitrypsin or alpha-2-macroglobulin [17, 18]. These protease inhibitors may interfere with the protein digestion step of the assay. In order to evaluate the impact of the patient’s inflammatory state on the accuracy of SRM-based protein quantification, we developed a SRM assay for the quantification of serum haptoglobin. Haptoglobin is a protein routinely measured in laboratory medicine, in particular for the detection of hemolytic anemia [19]. This SRM assay was then compared to a commercial immunonephelometric test used as reference quantification method. Finally, we determined whether the serum C reactive protein (CRP) concentration, a marker of the inflammatory state [20], had an impact on the haptoglobin SRM assay inaccuracy, which was defined as the bias between the LC-MS/MS and the immunonephelometric methods.

## Results and discussion

### Calibration of the haptoglobin SRM assay

**Figure 1:**
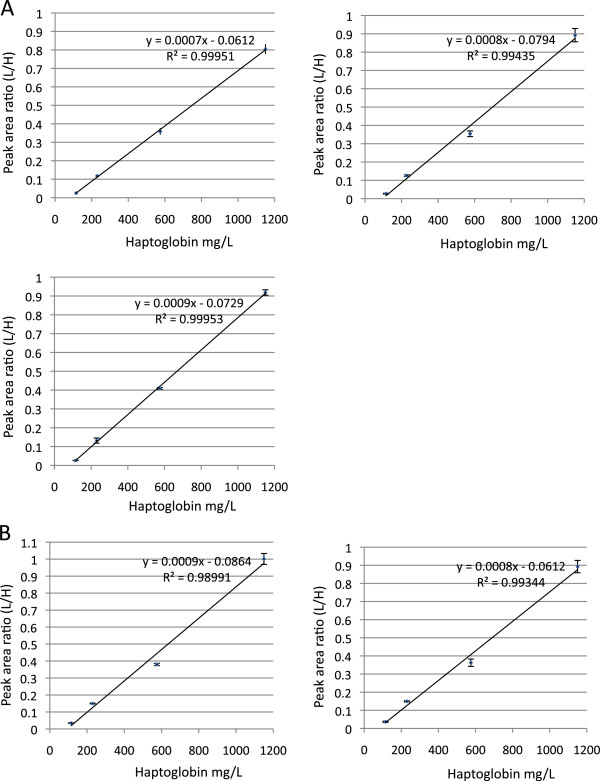
**Calibration of the haptoglobin SRM assay.** External calibration of the SRM assay was performed with the N Protein Standard SL (Siemens) used for the nephelometric assay calibration. Calibration solution was prepared according to the manufacturer’s instructions to a final concentration of 1150 mg/L. Dilutions were made in water at 575, 230, and 115 mg/L. A four points calibration was built ranging from 115 mg/l to 1150 mg/L. Each calibration point was injected in triplicate. **A)** Calibration curves obtained from three independent calibrations (different trypsin digestion) performed on the same day. **B)** Calibration curves obtained from two independent calibrations (different trypsin digestion) performed on different days.

Calibration of the SRM assay was performed using the commercial haptoglobin standard solution provided for the nephelometric immunoassay. A four points calibration was built ranging from 115 mg/L to 1150 mg/L. Precision of the calibration process was evaluated by comparing data from three independent calibrations (different trypsin digestion) performed on the same day. Calibration curves are presented in Figure 1A. CVs of peak area ratios obtained for the different calibration points ranged from 6.5 to 7.8%. Two additional calibrations were performed on two different days (Figure 1B). When including intra-day and inter-days five calibration data, the overall CVs of peak area ratios obtained for the different calibration points ranged from 6.4 to 17%. The highest CV was obtained for the lowest concentration of the calibration curve. These data demonstrated the reproducibility of the calibration process both intra- and inter-days.

### Haptoglobin quantification with the SRM assay

Haptoglobin concentration was measured by SRM in serum samples from 57 patients in two independent series. Two control sera were analyzed in triplicate in each series. CVs obtained (n = 6) were 12.9% at 698 mg/L for the N Protein Control SL Low (target value: 650 mg/L) and 11.8% at 1690 mg/L for the N Protein Control SL high (target value: 1740 mg/L). All results from patient samples were within the measuring range, except two sera with a haptoglobin concentration above 4600 mg/L and five samples with a concentration below 115 mg/L. Results are listed in Additional file 1.

### Comparison with the immunonephelometric assay

**Figure 2:**
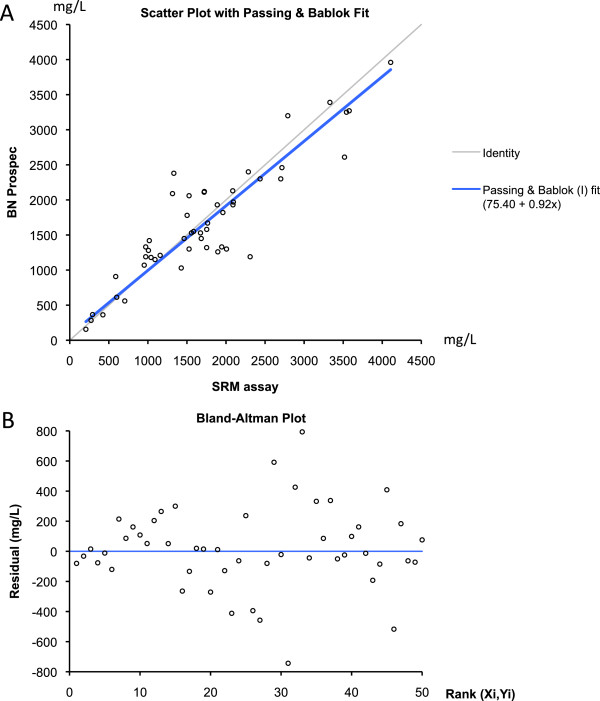
**Comparison of the haptoglobin SRM assay and the nephelometric immunoassay. A)** Passing-Bablok regression. **B)** Bland-Altman plot. Statistical analysis and graphics were made using Analyze-it V2.26.

Results from the SRM assay were compared with data of the commercial nephelometric immunoassay obtained on the same samples (Additional file 1). Passing-Bablok regression was performed using results within the measuring range of the two assays. A total of 50 values were included over a concentration range from 206 to 4108 mg/L (Figure 2A). The comparison indicates that the two methods provided overall similar results. The proportional bias was 0.92 (95% CI: 0.82 to 1.04) and the constant bias was 75.40 (95% CI: −71.09 to 251.04). Nevertheless, as shown on the Bland-Altman plot, important differences were observed for some samples between the two methods (Figure 2B).

Regarding samples not included in the Passing-Bablok analysis, discordant results between the SRM assay and the nephelometric immunoassay were obtained for only one serum. In this case, a haptoglobin concentration of 560 mg/L was found by immunonephelometry while it was below the limit of quantification (<115 mg/L) of the SRM assay. Otherwise, two samples measured above the measuring range of the nephelometric immunoassay (>4400 mg/L) were also found above the measuring range of the SRM assay (>4600 mg/L) and four samples measured below the measuring range of the nephelometric immunoassay (<75 mg/L) were also found below the measuring range of the SRM assay (<115 mg/L).

### Influence of inflammatory state of the patient on the SRM assay accuracy

**Figure 3:**
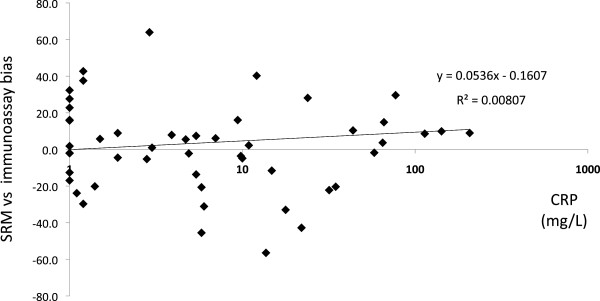
**Influence of inflammation on the haptoglobin SRM assay accuracy.** The bias between the SRM assay and the nephelometric immunoassay was calculated as follows: [haptoglobin SRM (mg/L) - haptoglobin nephelometry (mg/L)]/[mean (mg/L) of haptoglobin SRM and haptoglobin nephelometry] × 100. The serum CRP concentration was measured using a commercial turbidimetric immunoassay on an automated clinical chemistry analyzer. Graphic and linear regression analyses were made with Excel 2008.

Inflammation is associated with the release of various proteolytic enzymes, including elastin, cathepsins and matrix metalloproteinases, which act as important mediators of the inflammatory response [16]. In parallel to that, inflammation induces a strong increase of the serum level of various acute phase protease inhibitors, such as alpha-1-antitrypsin, alpha-1-antichimotrypsin or alpha-2-macroglobulin [17, 18]. One can hypothesize that these two events have an impact on the accuracy of SRM based protein assays. First, proteolytic enzymes may alter the *in vitro* stability of both endogenous protein/peptides and isotope-labeled internal standard peptides. Second, the serum concentration of acute phase protease inhibitors reaches the g/L range during inflammatory states and this may inhibit the trypsin digestion step of the SRM assay. We therefore investigated whether the extent of the bias observed between the haptoglobin immunonephelometric assay used as reference method and the LC-MS/MS assay was correlated to the inflammatory state of the patient. The bias was calculated using the following formula: [haptoglobin SRM (mg/L) - haptoglobin nephelometry (mg/L)]/[mean (mg/L) of haptoglobin SRM and haptoglobin nephelometry] × 100. Inflammatory state of the patient was determined by measuring the serum CRP concentration, which is the gold standard marker of inflammation in laboratory medicine [20]. Results of the linear regression analysis are presented in Figure 3. Samples yielding results out of the measuring range of one of the two assays were excluded from the analysis. The following linear regression equation was obtained: y (bias) = 0.0536 × (CRP) – 0.1607. Pearson correlation coefficient was 0.0898. As shown in Figure 3, some samples with CRP values within the normal range (<10 mg/L) were associated with a strong bias (>20%), either positive or negative, whereas the three samples with CRP values above 100 mg/L only presented minor bias (<10%). These data indicate that differences observed between results of the SRM and the immunonephelometric assays for some samples cannot be explained by analytical interferences related to the patient’s inflammatory state.

## Conclusions

A LC-MS/MS assay was developed for SRM quantification of haptoglobin in human serum samples. This assay yielded results comparable to those of a commercial immunonephelometric assay routinely used in clinical laboratories. We then demonstrated that the bias observed for some samples between the two analytical methods was not correlated to the serum level of CRP, a marker of inflammation. These data suggest that neither the release of proteolytic enzymes nor the increase in protease inhibitors level occurring during inflammation processes have a significant impact on the SRM assay accuracy. The differences observed between the two haptoglobin assays could thus be related to a combination of other factors, such as SRM assay imprecision and potential influence of other preanalytical variables [21]. Interferences affecting the nephelometric immunoassay accuracy may also be a possible explanation [3]. Regarding the possibility of generalizing the conclusion that inflammation does not significantly impact MS protein assay accuracy, two aspects must be distinguished. First, the stability of endogenous protein/peptides and heavy labeled internal standards in serum samples was shown to be species-dependent [15, 21]. Therefore, the sensitivity of this parameter to inflammation-associated peptidase/proteolytic activities must probably be evaluated for each SRM assay. In contrast, trypsin is the proteolytic enzyme used in the vast majority of bottom-up proteomic studies, including targeted protein quantitative assays. Our data suggest that this step of the sample processing is not sensitive to acute phase protease inhibitors present in serum samples. However, it is important to consider that this effect could vary depending on the trypsin digestion protocol used.

## Methods

### Chemicals

All chemicals were obtained from Sigma-Aldrich (Saint-Louis, MO), except water and acetonitrile from Romil (Cambridge, UK).

### Serum samples

Serum samples used in this study were collected at the University Hospitals of Geneva by experienced nurses in the context of standard patient care. The use of these human specimens was approved by the Ethical Committee for Research on Human Being of the Geneva state. Blood samples were collected on BD Vacutainer™ SST II Advance tubes and were centrifuged at 3’000 g for 5 minutes at room temperature upon reception at the laboratory. Measurement of the serum haptoglobin concentration was performed in the Division of Laboratory Medicine by immunonephelometry assay within 3 hours after centrifugation. The serum left over was stored at −80°C for MS analysis within two hours. Frozen sera were thawed and centrifuged at 3’000 g for 5 minutes at room temperature before MS analysis. Serum samples were submitted to a maximum of two freeze/thaw cycles.

### Analysis of haptoglobin by immunonephelometric assay

Haptoglobin was measured on a BN ProSpec nephelometer (Siemens, Marburg, Germany) using the N Antiserum Anti-Human Haptoglobin (Siemens) according to the manufacturer’s instructions. The assay had inter-assay CVs (n = 45) of 1.57% at 675 mg/L and 1.70% at 1394 mg/L. The limit of quantification was 75 mg/L. The reference range used at the Geneva University Hospitals for this assay is 412 to 1693 mg/L.

### Analysis of CRP by turbidimetric assay

CRP was measured on a UniCel DxC 880i clinical chemistry analyzer (Beckman Coulter, Brea, CA) using the Synchron CRP reagents (Beckman Coulter) according to the manufacturer’s instructions. The assay had inter-assay CVs (n = 45) of 4.44% at 8.7 mg/L and 2.76% at 37.2 mg/L. The limit of quantification was 1 mg/L. The reference range used at the Geneva University Hospitals for this assay is <10 mg/L.

### LC-MS/MS assay

#### External calibration and quality controls

External calibration of the SRM assay was performed with the N Protein Standard SL (Siemens) used for the nephelometric immunoassay calibration. Calibration solution was prepared according to the manufacturer’s instructions to a final concentration of 1150 mg/L. Dilutions were made in 25 mM aqueous ammonium bicarbonate (BA) at 575, 230, and 115 mg/L, divided into aliquots and frozen at −80°C until used. Each standard was injected in triplicate. In a few cases, one of the three replicates was removed from the calculation (relative error > 35% compared to the 2 other points).

Assessment of the SRM assay precision was performed using the High and Low N Protein Controls SL (Siemens) that were used for the nephelometric immunoassay.

#### Peptides selection

Two peptides from the haptoglobin beta chain were used in the SRM assay: VGYVSGWGR and VTSIQDWVQK (Additional file 2). These peptides were selected using the following process. Three aliquots of serum from a patient sample with a haptoglobin concentration measured at 2.66 g/L by immunonephelometry were digested as described in the Sample Preparation section. Digested samples were analyzed by RP-LC-MS/MS on a LTQ Orbitrap velos Pro (Thermo Electron, San Jose, CA, USA) equipped with a NanoAcquity system (Waters, Milford, MA, USA). Of the 13 peptides identified in common from the three samples, 7 were found to be proteotypic using SRMAtlas (http://www.srmatlas.org/), PeptideAtlas (http://www.peptideatlas.org/), and BLAST (http://blast.ncbi.nlm.nih.gov/Blast.cgi). The two peptides used in the SRM assay were selected from this list of proteotypic peptides based on the following criteria: reproducibility of the retention time, peak shape, absence of matrix interferences, limit of detection, calibration curve linearity, and the consistency of the collision energy for peptide fragmentation. Isotope-labeled peptides used as internal standards were obtained from JPT Peptide Technologies (Berlin, Germany) (Additional file 2). Lyophilized heavy peptides (26 nmol) were dissolved in 100 μl of 5% acetonitrile (ACN), 0.1% formic acid (FA). A volume of 5 μL of this stock solution was diluted then in 200 μL 5% ACN, 0.1% FA.

#### Sample preparation

A volume of 5 μl of calibrator, control or serum sample was mixed with 45 μl of 25 mM BA. Reduction, alkylation of cysteines, and trypsin digestion were performed using a protocol adapted from Proc *et al.* [22], as described below. The mixture was further diluted with 238.6 μl of 25 mM BA. A volume of 32.1 μl of 50 mM tris (2-carboxyethyl)phosphine (TCEP) was added and the mixture was incubated 30 min at 60°C. Then, 35.6 μl of 100 mM iodoacetamide was added and the mixture was incubated 30 min in the dark at 37°C. At the end of the incubation, 100 μl of methanol were added followed by 43.8 μl of a trypsin solution at 0.4 mg/ml. Digestion was performed at 37°C for 4 h and stopped with 80 μl of 0.1% FA. After evaporation, samples were desalted using a C18 Macro SpinColumn (Harvard apparatus, Holliston, MA). After sample loading, the column was washed twice with 5% ACN, 0.1% FA and elution was performed twice with 150 μL of 50% ACN, 0.1% FA. The flow-through was dried under vacuum, dissolved in a volume of 200 μL 5% ACN, 0.1% FA and 5 μL of diluted heavy peptides was added.

#### LC-MS/MS analysis

HPLC separation was performed on an Accela system (Thermo-Scientific, Waltham, MA) using a Hypersil GOLD Guard 10 × 1 mm 3 μm particle precolumn (Thermo-Scientific) and a Hypersil GOLD C18 100 × 1 mm 1.9 μm particle column (Thermo-Scientific). Mobile phases used for the analysis were: A) 0.2% FA in water (v/v) and B) 0.2% FA in ACN (v/v). Sample (10 μL) was injected into the HPLC system at an initial condition of 5% B during 1 min and rising to 30% after 14 min. The column was then washed at 100% B for 1 min before returning to starting conditions in 7 min. A constant flow rate was set at 80 μL/min and the column compartment was set at a constant temperature of 50°C. The HPLC system was hyphenated to TSQ Quantum Access triple stage quadrupole equipped with a heated electrospray ionization probe (Thermo-Scientific). Peptides were quantified using SRM in positive ion mode under the following conditions: ionization voltage, 3500 V; sheath gas pressure, 10 arbitrary units; auxiliary gas pressure, 1 arbitrary units; capillary temperature, 270°C; Tube lens offset, 92; Collision pressure, 1.5. Two proteotypic peptides were analyzed in the assay: VGYVSGWGR was used for the quantification of haptoglobin and VTSIQDWVQK was used as verifier. Examples of extracted ion chromatograms for these two peptides are presented in Additional file 3. We determined peak areas for the endogenous and internal standard heavy peptides in Xcalibur 2.1.0.1139 (Thermo-Scientific). Three transitions were used per peptide (Additional file 2). The transitions per peptide or internal standard were summed. The response of each peptide was calculated as the ratio of the peak area of the endogenous peptide to the peak area of the corresponding heavy peptide used as internal standard. Patient samples with a haptoglobin concentration greater than the highest standard (1150 mg/L) were reanalyzed after dilution in 25 mM BA. A maximum dilution of ¼ was used so that the maximum concentration measured with the haptoglobin SRM assay was 4600 mg/L.

### Statistics

All graphics and statistics were made with Excel 2008, except Passing-Bablok regression [23] and Bland-Altman plot [24], which were made using Analyze-it V2.26.

## Authors’ information

OL is chemist at the Clinical Proteomics Laboratory of the Geneva University Hospitals; DH is head of the department of Genetic and Laboratory Medicine at the Geneva University Hospitals; PL is head of the Clinical Proteomics Laboratory and of the Toxicology and Therapeutic Drug Monitoring Laboratory at the Geneva University Hospitals.

## Electronic supplementary material

Additional file 1: **Haptoglobin concentrations, CRP concentration, and bias values obtained for individual serum samples.** (PDF 40 KB)

Additional file 2: Table S1: Transitions used in the haptoglobin SRM assay. (PDF 52 KB)

Additional file 3: **Supplementary Figure 1.** Extracted ion chromatograms of the VGYVSGWGR peptide (quantifier) and the VTSIQDWVQK peptide (verifier). (PDF 916 KB)

## Abbreviations

LC-MS: Liquid chromatography coupled to tandem mass spectrometry
SRM: Selected reaction monitoring
CRP: C reactive protein
BA: Ammonium bicarbonate
ACN: Acetonitrile
FA: Formic acid
TCEP: Tris (2-carboxyethyl)phosphine.
