# Supplementary material for: Assessment of the influence of the patient’s inflammatory state on the accuracy of a haptoglobin selected reaction monitoring assay
Source: Clin Proteomics. 2014 Nov 1;11(1):38. doi: 10.1186/1559-0275-11-38 (PMC4228078; doi:10.1186/1559-0275-11-38)
Supplement: Supplementary file 1 — Additional file 1: Haptoglobin concentrations, CRP concentration, and bias values obtained for individual serum samples. (PDF 40 KB) [file 12014_2014_80_MOESM1_ESM.pdf]

	Haptoglobin concentration (mg/L)			
Serum samples	LC-MS/MS assay	Immunonephelometric assay	Bias	CRP (mg/l)
16-169	1502	1780	-16.9	1
16-70	1018	1420	-33.0	17.8
17-125	1585	1550	2.2	10.9
17-129	1464	1450	1.0	3
17-170	> 4600	> 4450		161
17-172	2716	2460	9.9	142.2
17-32	972	1190	-20.2	1.4
17-33	1895	1260	40.3	12.1
17-37	3517	2610	29.6	77.1
17-42	3330	3390	-1.8	58
17-55	1683	1450	14.9	66
17-66	1315	2090	-45.5	5.8
17-75	3542	3250	8.6	113.7
18-172	2006	1300	42.7	1.2
18-2	1945	1330	37.6	1.2
18-20	1007	1280	-23.9	1.1
18-3	1427	1030	32.3	<1.0
18-68	1752	1320	28.1	23.9
18-98	1157	1210	-4.5	1.9
19-197	1720	2110	-20.4	34.7
20-13	2286	2400	-4.9	10
20-14	2701	2300	16.0	9.4
20-7	4108	3960	3.7	64.9
22-101	> 4600	> 4400		67
22-116	<115	<75		1.9
22-17	2436	2300	5.7	1.5
22-197	972	1330	-31.1	6
22-2	1673	1530	8.9	1.9
22-20	1723	2120	-20.7	5.8
22-204	<115	<75		14.1
22-4	1888	1930	-2.2	4.9
22-5	2791	3200	-13.7	5.4
22-62	1527	2060	-29.7	1.2
23-17	1960	1820	7.4	5.4
23-198	1764	1670	5.5	4.7
23-5	1558	1530	1.8	<1.0
24-139	3577	3270	9.0	207
24-157	2309	1190	64.0	2.9
24-49	2086	2130	-2.1	<1.0
24-51	1040	1180	-12.6	<1.0
24-67	1753	1580	10.4	43.7
24-68	< 115	<75		1.1
24-69	588	908	-42.8	22
24-70	953	1070	-11.6	14.8
25-124	601	613	-2.0	1
25-157	704	560	22.8	<1.0
25-165	424	362	15.8	<1.0
25-18	206	156	27.6	<1.0
25-186	<115	<75		14.1

25-193	1527	1300	16.1	1
25-3	1091	1150	-5.3	2.8
25-4	2089	1930	7.9	3.9
25-56	273	283	-3.6	9.8
25-6	<115	560		<1.0
25-64	1332	2380	-56.5	13.7
25-70	2094	1970	6.1	7
25-87	292	365	-22.2	31.8
