# Supplementary material for: Assessment of the influence of the patient’s inflammatory state on the accuracy of a haptoglobin selected reaction monitoring assay
Source: Clin Proteomics. 2014 Nov 1;11(1):38. doi: 10.1186/1559-0275-11-38 (PMC4228078; doi:10.1186/1559-0275-11-38)
Supplement: Supplementary file 2 — Additional file 2: Table S1: Transitions used in the haptoglobin SRM assay. (PDF 52 KB) [file 12014_2014_80_MOESM2_ESM.pdf]

Supplementary Table 1

Transitions used in the haptoglobin SRM assay. CE: collision energy.

Peptide	Position in the UniProt sequence (P00738)	Position in the haptoglobin beta chain sequence	*m/z*	Fragment 1	CE, eV	Fragment 2	CE, eV	Fragment 3	CE, eV
VGYVSGWGR	278-286	117-125	490.75	475.24(y4)	16	562.27(y5)	16	661,34(y6)	17
VGYVSGWGR*			495.75	485.25	16	572.28	16	671,35	17
VTSIQDWVQK	392-401	231-240	602.34	675.35(y5)	21	803.41(y6)	6	1003.52(y8)	21
VTSIQDWVQK*			606.32	683.34	21	811.40	6	1011.52	21
