# Supplementary material for: Assessment of the influence of the patient’s inflammatory state on the accuracy of a haptoglobin selected reaction monitoring assay
Source: Clin Proteomics. 2014 Nov 1;11(1):38. doi: 10.1186/1559-0275-11-38 (PMC4228078; doi:10.1186/1559-0275-11-38)
Supplement: Supplementary file 3 — Additional file 3: Supplementary Figure 1. Extracted ion chromatograms of the VGYVSGWGR peptide (quantifier) and the VTSIQDWVQK peptide (verifier). (PDF 916 KB) [file 12014_2014_80_MOESM3_ESM.pdf]

VGYVSGWGR calibrator 1150 mg/L

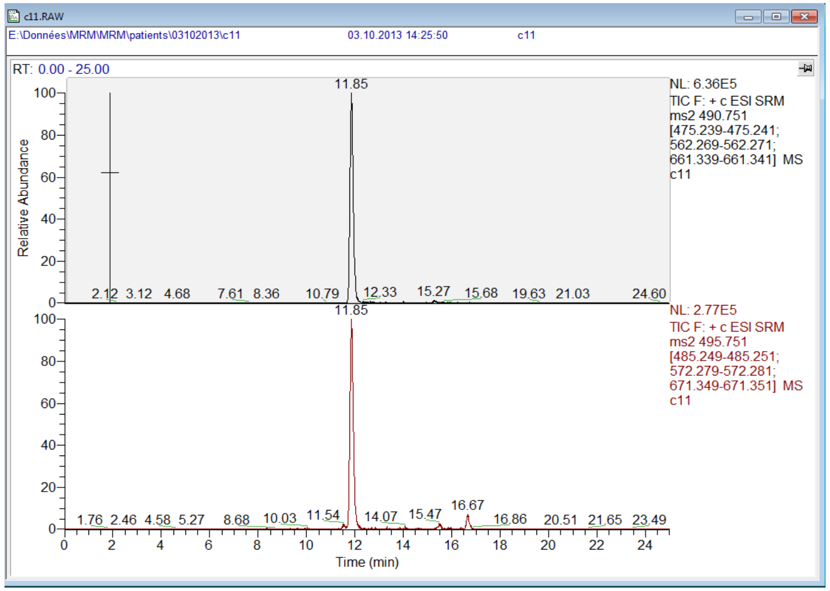

VGYVSGWGR calibrator 115 mg/L

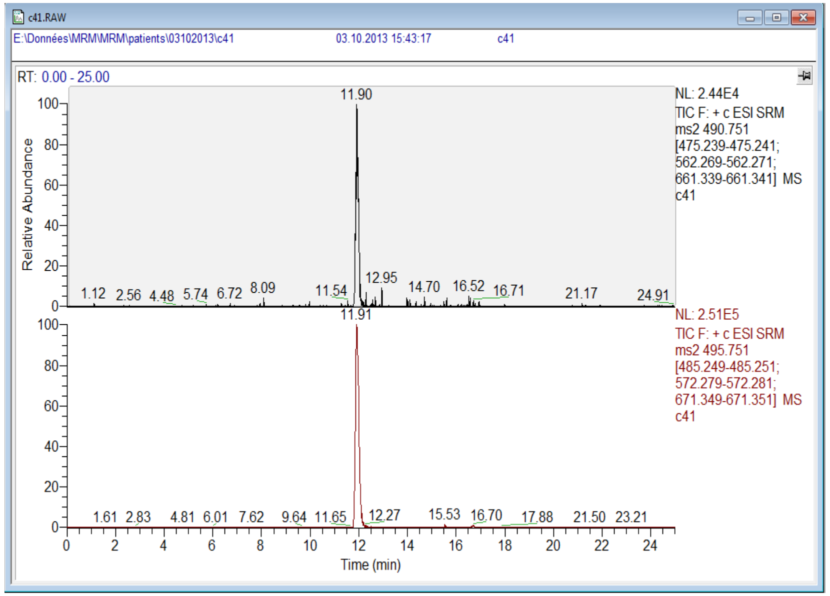

VGYVSGWGR patient sample at 362 mg/L.

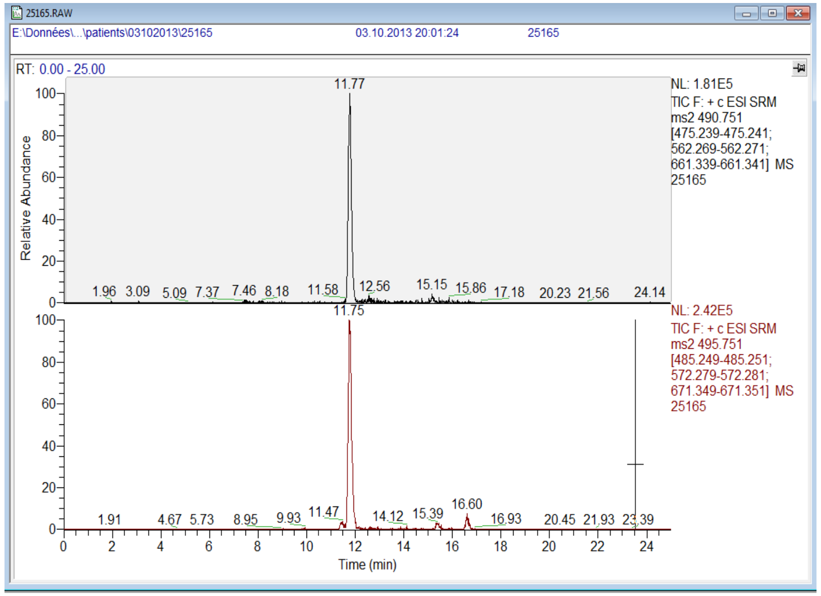

VTSIQDWVQK patient sample at 1560mg/L.

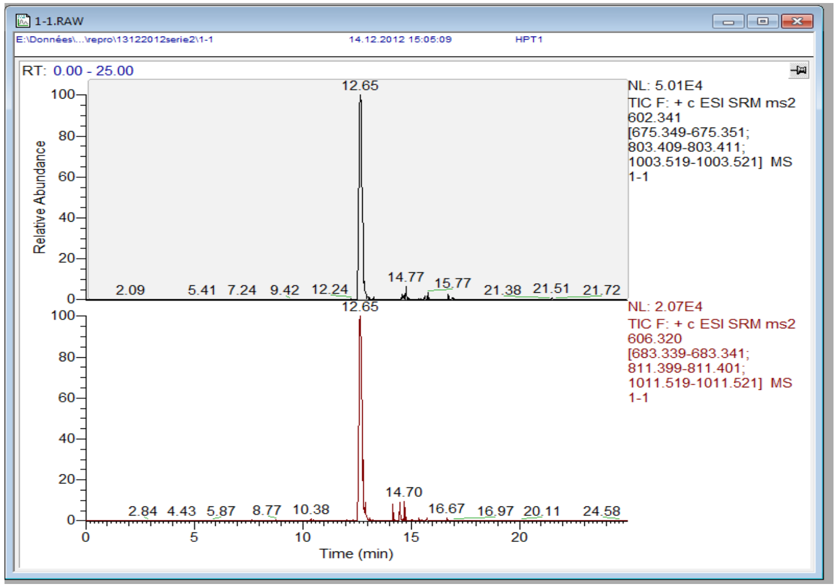

Supplementary Figure 1: Extracted ion chromatograms of the VGYVSGWGR peptide (quantifier) and the VTSIQDWVQK peptide (verifier).
